# Correction: Barriers and facilitators to implementing six monthly multi-month dispensing of antiretroviral therapy in two urban HIV clinics during the COVID-19 Era in Malawi

**DOI:** 10.1371/journal.pgph.0004203

**Published:** 2025-01-15

**Authors:** The PLOS Global Public Health Editors

## Notice of Republication

This article [1] was republished on January 3, 2025, to address a file error. The publisher apologizes for the error. Please download this article again to view the correct version.
