# Supplementary material for: Barriers and facilitators to implementing six monthly multi-month dispensing of antiretroviral therapy in two urban HIV clinics during the COVID-19 Era in Malawi
Source: PLOS Glob Public Health. 2024 Dec 31;4(12):e0003900. doi: 10.1371/journal.pgph.0003900 (PMC11687738; doi:10.1371/journal.pgph.0003900)
Supplement: Attachment — Submitted filename: The_Rebuttal letter_15_Oct_24.pdf [file pgph.0003900.s002.pdf]

Dear Editor,

We appreciate your valuable feedback and the opportunity to address the comments from the reviewers. Please find below a point-by-point response to the queries raised.

PLOS Review feedback

Reviewer #1: Summary:
A cross-sectional study done in two high volume urban health facilities in Malawi using qualitative and quantitative data to review barriers and facilitators of six monthly multi month dispensing of antiretroviral drugs during COVID 19 era. Major barriers to implementation were low drug supplies and lack of understanding of policy guidance. The author recorganizes that implementation of 6-MMD may have led to cases of delayed or missed clinic appointments and missed opportunities for viral load sample collection if clinic appointments were not aligned. Major facilitators of 6-MMD were health care workers orientation and review meetings, teamwork among staff, and use of the electronic medical records system to track patients. Additional motivators to implementing 6-MMD included reduction in the number of patient visit and reduced workload at health facilities. Study findings may be relevant in the development and implementation policies on multi month dispensing of ART among HIV positive clients.

Overall comments
Query 1: Review document and remove any double spaces.

Response 1: All the double spaces have been removed.

Query 2:- Review In text referencing for consistency. E.g …..visits(3). …… facilities. (5, 6) …….. patients (7). care, (12, 13) (14, 15),

Response 2: This has been corrected throughout the document.

Query 3: - Minor spelling errors

Response 3: We have corrected spelling errors throughout the document**.**

Query 4: Title: Barriers and facilitators to implementing six monthly multi-month dispensing of antiretroviral therapy in HIV clinics during the COVID-19 Era in Malawi
Comment: Title seems to suggest that findings are generalizable to whole population while this was a descriptive study limited to two high volume health facilities in an urban setting. Please review

Consider: Barriers and facilitators to implementing six monthly multi-month dispensing of

antiretroviral therapy in two urban /selected HIV clinics during the COVID-19 Era in Malawi.

Response 4: This change has been made. Please see updated title on Line 1 to Line 2

Query 5:. Abstract:
Summary is well presented and includes key findings from the study.
a. Conclusion section needs review: Looks like a repetition of the findings. Consider revising and include only key findings from the study. Avoid "commanding" language e.g … should… Suggestion: Major facilitators to transition to 6-MMD included health care worker capacity building, teamwork and use of electronic medical records while major barriers included low drug supplies and lack of understanding of policy guidance. These findings may be helpful when developing strategies for increasing coverage and uptake of 6-MMD of ART"

Response 5: This correction has been made. Please see updated abstract from Line 57 to Line 60

Query 6:3. Introduction
a. Include information/background on the two pilot facilities: Number of PLHIV on treatment, Changes in number of clients on 6-MMD...…… This may help put the narratives into context

Response 6: We have added information about the number of PLHIV and change of clients on 6-MMD in order to put the narrative in context. At Mpemba Health Centre about 78% (320/410) of the patients had been transitioned to 6-MMD while at Limbe Health Centre 80% of the patients had been transitioned to 6-MMD at the time of the study. Please see Line 122 to Line 124

Query 7:b. ……Decreased rate of transmission and longer quality of life….. Comment: Consider rewording to …"Decrease in HIV transmission rate and improved quality of life"….

Response 7:This change has been made. Please Line 67

Query 8:c. Suppressed viral load
Comment: Please include reference value (? <50 copies/ml ?200 copies/ml ?<1000 copies/ml)

Response 8: The reference value of < 1000 copies has been inserted. Please see Line 92

Query 9:d. In April 2020 following the inception of the COVID-19 outbreak, the eligibility criteria for 6-MMD were revised to allow patients receiving dolutegravir-based ART regimens who weighed more than 20 kilograms and had been on ART for at least 3 months to receive 6-MMD (8)

Comment: Did this include both those newly initiated on DTG and those already on ART but transitioned to DTG based on availability of new molecules? Please clarify/add

Response 9: This included both newly initiated on DTG and those that were already on ART and had transitioned to DTG. The scale-up of DTG was based on availability of new molecules but was also the initiative of the government to provide more efficacious regimens to patients.

Query 10 :e. With the relaxed policy, patients did not have to have a suppressed viral load, also those with adherence problems were not contra-indicated to receive the 6-MMD (8) Comment Consider including a statement that although high VL and patient adherence challenges were not a contraindication to 6-MMD, adequate support systems were put in place to ensure patients achieved viral suppression.

Response 10: This is a good suggestion. However, this is more of an assumption. The policy guidance did not emphasize on putting adequate support systems in order for patients to achieve viral suppression during the peak of the COVID-19 pandemic**.**

Query 11:4. Methods: I request that the section be reviewed by a subject matter expert preferably a behavioral scientist with support from a statistician.

Response 11: This manuscript has been reviewed by several esteemed scientists. The second author Dr Agatha Bula is behavioral scientist. The Senior Authors Dr Mitch Matoga and Dr Thulani Maphosa who have conducted similar studies to this, also reviewed the manuscript.

Query 12:5. Results: I request review by subject matter expert preferably a behavioral scientist with support from a statistician

Response 12: This manuscript has been reviewed by several esteemed scientists. The second author Dr Agatha Bula is behavioral scientist. The Senior Authors Dr Mitch Matoga and Dr Thulani Maphosa who have conducted similar studies to this, also reviewed the manuscript

Query 13:a. Question: Some countries recommend 6 monthly clinic consultation, but 3 monthly ART pick up by clients. It looks like policy guidance in this case was slightly different. Please confirm.

Response 13: This policy guidance was different. Patients had 6 monthly consultations during which they had a clinical assessment and had ART pick pick-ups. Thus they had 6 monthly consultations with 6 monthly pick-ups.

Query 14 :b. Consider adding absolute numbers to the narrative given that the absolute numbers (Ref Table 2) are small, to put the percentages into context for the reader. E.g 96% (n=xx).

Response 14: This has been done. See the narrative from Line 206 to Line 215

Query 15:c. Minor typing errors: Loss of follow up: Comment: Did you men lost to follow up?

Response 15: Yes, I meant lost to follow up. The typing error has been corrected. Please see Line 212 and Table 2

Query 16:d. Table 2: Transitioning people to 6-monthly refill has led to unstable patients e.g people with high HIV RNA viral load being provided 6 months' supply which would not have been the case before
Consider adding in discussion section that despite policy guidance that allowed 6-MMD for all clients irrespective of viral load test result among others, one would have expected most respondents to agree with the statement which was not the case. Either previous guideline may not have been followed as required and a few unstable patients were getting 6-MM or respondents did not understand the question.

Response 16: This has been added in the discussion. Please see Line 468 to Line 474

Query 17:6. Discussion: Additional inputs from a behavioural scientist would be helpful

Response 17: This manuscript has been reviewed by several esteemed scientists. The second author Dr Agatha Bula is behavioral scientist. The Senior Authors Dr Mitch Matoga and Dr Thulani Maphosa who have conducted similar studies to this, also reviewed the manuscript

Query 18:a. …….. Dispensing of drugs to clients who were not eligible for 6MMD such as those with high VL……
Comment: In the introduction section, it is stated that patients did not need to have a suppressed viral load to receive 6-MMD. The two seem to contradict each other. Please review.

Response 18: We have edited that sentence and have discussed that contradiction. Please see Line 468 to 474

Query 19:b. Most references are based on studies done in Malawi. Review of study findings from other countries may enrich the discussion.

Response 19: We included studies from other countries including South Africa, Mozambique and Haiti. Please see Line 460 and Line 511

The additional reference added are :

Parrish C, Basu A, Fishman P, Koama JB, Robin E, Francois K, et al. Estimating the effect of increasing dispensing intervals on retention in care for people with HIV in Haiti. EClinicalMedicine. 2021;38:101039.

Saura-Lazaro A, Augusto O, Fernandez-Luis S, Lopez-Varela E, Fuente-Soro L, Bila D, et al. HIV care retention in three multi-month ART dispensing: a retrospective cohort study in Mozambique. AIDS. 2024;38(9):1402-11.

Query 20:c. For consideration in discussion: Increase in number of clients not keeping clinic appointments may either be attributed to clients having more drugs or fear of coming to health facilities due to COVID 19. It is not clear from the discussion where there was an apparent increase in number lost to follow up. It could be true that there was an increase in number LTFU or what is a reporting issue. Additional literature may be helpful.

Response 20: We have added this in the discussion section. Please see Line 510 to Line 513. Though negative effects, such as the delay in patients reporting to the health facility for clinic visits leading to their misclassification as lost to follow-up, have also been reported. However, contrary to this health care workers noted that they felt that there was an improvement in retention into care at the facility. Studies done in Mozambique and Haiti have also shown than provision of ART for longer intervals were associated with improved retention into care and not loss to follow up.

Query 21: a. Drug packaging. …… Other studies have shown that providers are concerned about patients having challenges in collecting many bottles and storing them (12, 16). Comment: Consider including client perspective in the discussion. Looks like in the two quoted references, responses from beneficiaries was slightly different" …. They also expressed concerned about clients' challenges with ART storage at home, but clients reported no storage problems Ref 12. …….. Contrary to key informant concerns about patients' responsibility to manage larger quantities of ART, patients receiving 6-month refills were highly motivated and did not face challenges transporting, storing or adhering to treatment (ref 16).

Response 21: We have added that to the discussion section. Please see Line 503 to Line 504

Query 22: Our study suggests that packaging of drugs for several months in one bottle should considered when implementing MMD
Comment: Consider rewording to … Our study suggests that packaging of drugs for several months in one bottle may be beneficial when considering implementation of MMD (Let us avoid commanding language)

Response 22: This has been done. Please see Line 504 to Line 506

Reviewer #2: I congratulate the authors for this perspective on how COVID-19 was intersecting with HIV care and retention, however, here are some minor revisions:

Abstract
Query 23:1. "the six-monthly multi-month dispensing (6-MMD)" - The term "six-monthly multi-month" seems redundant. "Six-monthly dispensing (6-MMD)" should be sufficient.

Response 23: The wording is in line with the literature which commonly uses multi-month dispensing and abbreviates it as MMD. We propose to still maintain the wording of multi-month dispensing.

Query 24:2. "from 13 January 2022 to 5 February 2022" - Consistency in date format is important. It would be clearer to use either "January 13, 2022" or "13 January 2022" throughout the document.

Response 24: We have used 13 January 2022 format through out the document

Query 25.3. "conduction of orientation meetings" - The correct term should be "conducting orientation meetings."

Response 25: This has been corrected throughout the document.

Methodology and discussion section

Query 26:1. "Orientation and review meetings, teamwork among staff, and the use of the electronic medical records system" instead of "of orientation and review meetings, teamwork among staff and use of the electronic medical records system"- Corrected the phrasing for clarity and grammatical correctness.

Response 26: This has been corrected. See Line 52. Line 528 to Line 529**.**

Query 27:2. "It was cited as another motivator" instead of "were cited as other motivators" - Corrected subject-verb agreement.

Response 27: This has been corrected. Please see Line 54 to Line 55

Query 28:3. "Conducted in Malawi have shown"instead of "conducted in Malawi have showed" - Corrected verb tense.

Response 28: This has been corrected **.** Please see Line 462

Query 29:4. "For those requiring three-monthly MMD, community ART, or fast-track refills due to limited healthcare workers' knowledge of the requirements" instead of "those requiring three-monthly MMD, community ART, or fast-track refills because limited healthcare workers' knowledge of the requirements" - Corrected phrasing.

Response 29: This has been corrected. Please see Line 476 to Line 477

Query 30 : 5."Vaccine was required to be administered when attending a clinic visit" instead of "vaccine was required to be administered when attending a clinic visit is a new finding" - Corrected tense for clarity.

Response 30: The authors cannot see where the change is required.

Query 31:6."For other patients outside of the HIV arena"instead of "other patients outside of the HIV arena"- Corrected preposition usage.

Response 31: I have edited the sentence. Please see Line 495 to Line 497

Query 32: 7."Classified as lost to follow-up"instead of "being classified as lost to follow-up"- Corrected phrasing for conciseness.

Response 32: I have edited the sentence. Please see Line 509

Query 33:8."Nevertheless, our results can guide decision-makers" instead of "Nevertheless, our results can guide decision makers"- Corrected punctuation.

Response 33: This has been corrected. Please see Line 523 to Line 524

Reviewer #3: The paper written by Chamanga et. al evaluated barriers and facilitators for providing six months duration antiretroviral therapy (ART) to people living with HIV (PLHIV) in Malawi at the peak of the COVID-19 pandemic. They also looked at the health care worker perspective on the effects of six-monthly refills on the health system and clients. The authors employed a mixed methods approach through a survey and key informant interviews to ten types of health care cadres working closely with PLHIV on ART at two health facilities in early 2022.
The paper is well written and easy to comprehend. While the survey also looked at the effects of 6-MMD (Table 2), these findings are not reflected in the title or the conclusion of the paper.
Specific comments for different sections are as follows:
Introduction

Query 34:1. Reference 8, maybe used in conjunction with Edition 5, 26th January 2021 where routine viral load testing was suspended except for the 6 months point and targeted. (https://dms.hiv.health.gov.mw/dataset/covid-19-guidance-for-hiv-services-edition-5/resource/5782288a-dfaf-43d5-8fb8-348c3d11fa33)

Response 34: That is correct. We have added it as a reference.

Query 35:2. Introduction and discussion to include mention of global, regional and local context. It does not come out very clearly except if one goes to check references where there is then mention of Africa, CQUIN, Indonesia, South Africa, PEPFAR supported countries etc. May be interesting to know 6-MMD coverage for the study sites at that point in time if information available.

Response 35: We have included mention of the global, regional and local context**.** Please Line 72 to Line 82 of the introduction section**.** The 6-MMD coverage at the Limbe Health Centre was around 80% and Mpemba Health Centre was 78%. Please see Line 122 to Line 123

We have added the following reference while updating this section:

World Health Organisation. Consolidated guidelines on the use of antiretroviral drugs for treating and preventing HIV infection: recommendations for a public health approach. 2016.

Mantell JE, Zech JM, Masvawure TB, Assefa T, Molla M, Block L, et al. Implementing six multi-month dispensing of antiretroviral therapy in Ethiopia: perspectives of clients and healthcare workers. BMC Health Serv Res. 2023;23(1):563.

Query 36:3. Page 7, 1st paragraph states that "However, the impact of scaling-up the 6-MMD strategy has not been evaluated during the COVID-19 pandemic in Malawi. We evaluated the barriers and facilitators of implementing the 6-MMD strategy to better understand its impact during the COVID-19 pandemic among PLHIV receiving care at HIV clinics in Malawi." It may be useful to further clarify "impact".

Response 36: Thank you for this comment. We have re-worded it to implementation. We wanted to evaluate the barriers and facilitators in implementing the 6-MMD. Please see Line 108

Methods

Query 37:1. The study population definition statement is inclined towards the sampling or inclusion criteria. I suggest a revision.

Response 37: We have revised the statement to describe that they had to be health care workers working in the two HIV care clinics. Please see Line 126 to Line 127

Query 38:2. Is it possible to also have the total number of HCWs who were working in the ART clinics to show how the sample of 77 was decided upon. The selected cadres, what are their roles in the provision of 6-MMD?

Response 38: The total number of health care workers in the ART clinic of in Limbe and Mpemba Health Centre during the time period were about 100. The sample of 77 was based on convenience. We approached all health care workers at the health facility who were available to take the survey. Other health care workers may not have been available as they were not on duty. The clinician or nurse dispenses the medication. The ART clerk enters the data of the patient in the Electronic Medical Records System (EMRS). The HIV Diagnostic Assistant is responsible for conducting HIV test or viral load testing. The Adherence Support Officer provides adherence counselling. The Youth Champion provides peer support to adolescents living with HIV ages 10-24 years. The Expert Client provides peer support to adults living with HIV. Health Surveillance Assistant conducts community-related activities. The pharmacy assistant keeps stock of the ART supply

Query 39:3. Are there health care workers who are below the age of 18?

Response 39: There is a cadre of Youth Champions who are adolescents living with HIV aged who function as peer educators and counsellors of fellow adolescents between ages of 10-24 years. However, this cadre has to be at least 18 years of age, therefore we have removed that description from the narrative.

Query 40:4. Key informant purposive selection: was there any special reason why 100% of clinicians from the survey went on to do the key informant interviews, rather than proportionate apportionment of the cadres? Also, in the survey 10 types of cadres participated but for key informant interviews only 7 types of cadres were included.

Response 40: It was not 100% of the clinicians who took part in the survey, that took part in the interview. Two of the clinicians took part only in the key informant interviews but not the survey. There were more clinicians because the cadre such as the health-facility-in charge was a clinician. The prescriber was also a clinician. Indeed, we did not have any ASO, psychosocial counsellor or pharmacists in the key informant interviews particularly due to lack of their availability.

Query 41:5. The framework analysis, sentence number 4: Clarify the attitude of affected patients regarding the policy since patients were not part of the study. Also, the patients' part does not appear in the abstract.

Response 41: Thank you for the comment. We have removed that part since patients were not part of the study

Query 42:6. How many tablets had ODK loaded? How many locations within health facilities had tablet stations for questionnaire filling?

Response 42: Four tablets had ODK loaded. The tablets were with the research assistants who moved around in the ART clinics identifying possible participants for the interview. Once a participant was identified, they were provided the tablet and a quiet space was identified where they could answer the questions. The quiet spaces included the consultation room or the patient waiting area as the interviews were conducted after most patients had gone home.

Query 43:7. Consent is not mentioned for key informant interviews

Response 43: Written consent was required for the key informant interviews. It has been mentioned in Line 163

Query 44: 8. Is there a specific reason why key informant interviews where only done in Chichewa, yet the survey question had English and Chichewa options?

Response 44: They key informant interviews were conducted in the native language of Chichewa to allow participant to fully express themselves. The survey had Chichewa and English options as the questions were mostly structured and just required ticking.

Query 45:9. Ethical considerations: informed consent mode? written/verbal

Response 45: The informed consent mode was written. His has been specified in the manuscript. Please see Line 196

Results:

Query 46: 1. Expert clients: in addition to being HCWs, they double up as patients. Could a sub-analysis of this sub-population have any significant finding?

Response 46: The primary role of expert clients in our setting is to provide peer-to-peer adherence support to patients in consuming ART drugs. As this paper was not focused solely on adherence but the dispensation of drugs, their sub analysis would not change the findings markedly.

Query 47:2. Table 2 results are not included in the discussion or conclusion

Response 47: We have added a narration in the discussion. **Please see line 467 to Line 474.**The results in Table 2 are also in agreement with what was in the key informant interviews.

Query 48: 3. Were there any significant differences in responses by gender and by cadre?

Response 48: We did not conduct this analysis. A variation in the responses in gender was not the primary interest of the study.

Query 49:4. Table 3: Row 12, add (SD) after mean age

Response 49: This has been added

Query 50:5. Table 3: Row 13, mean years worked is presented as a range (1-5years)

Response 50: This question was a categorical question. I have added the additional categories and the frequency.

Query 51:6. Table 4: Cotrimoxazole not mentioned, only ART drugs mentioned compared to information on the previous page

Response 51: This has been added

Query 52:7. Table 4: Drugs packed in 1 bottle for 3 months vs a bottle with 6-month supply on previous page. Need to align.

Response 52: That error has been corrected. It is packaging drugs for 3 months in one bottle. Please see line 242

Query 53:8. Table 4: HCW vs patient factors/barriers/facilitators – classification and presentation

Response 53: As the study included only Health Care Workers. We can only speak strongly on the health care worker barriers or facilitators and so there is no need for classification.

Query 54:9. Table 4: Orientation and review meeting description: May need additional information for review meetings

Response 54: This information has been added. Review meetings helped to track the progress in implementation of the policy. Please see Table 4

Query 55:10. Table 4: Prior MMD experience: for HCWs or health facilities?

Response 55: This is at the facility and not for the individual health care worker.

Query 56:11. Table 4: Improved retention into care: 'The reduced patient visits ensured that patients came on the specified date and improved retention of the patients'. Description may need review

Response 56: The description has been edited. The patients were more likely to come on the specified date if visits were more spaced out and this improved retention of the patients. Please see Table 4

Query 57:12. Table 4: Delayed or missed patient visits: The assumption is that the appointment are for other services

Response 57: The appointment was for refilling of the ART drugs and clinical review. I have added this in the Table 4

Query 58:13. Table 4: Apart from reduced patient visits saving patients money, what were other benefits to patients?

Response 58: This benefit was highlighted by the health workers. However**,** as we did not include patients in the study population, we can not say much about other benefits of the 6-MMD policy to them.

Query 59:14. May consider including literature on policy for Cotrimoxazole preventive therapy (CPT) for stable patients, Edition 5 of 26th January 2021 also mentions 6MMD policy for ART during stock outs for CPT

Response 59: We have included this literature in the references.

Query 60:15. Page 15, 6th paragraph quotation and page 18: 6 months in 1 bottle, vs 3 months, vs 30 tablets vs type of dolutegravir based regimen vs 90 tablets in a bottle - alignment of HCW knowledge

Response 60: It is 3 months' supply in one bottle. The 3-month supply is about 90 tablets**.** The 13A is the Dolutegravir regimen. It comprises of Tenofovir/ Lamivudine / Dolutegravir in a combine fixed tablet.

Query 61:16. In the methods section the authors state that "The questionnaire had a combination of structured questions and a three-point Likert scale (agree, neutral, disagree), and focused on questions on perceived barriers and facilitators of implementing the 6-MMD strategy". Is table 2 reflective of the structured questions or there are other results/questions for this part?

Response 61: Table 2 is reflective of the structured questions. I have re-worded the description to "experiences of conducting the 6-MMD" as I note that it does not explicitly focus on barriers and facilitators only.

Discussion:

Query 62:1. Some themes in Table 4 are evidence of limited HCW knowledge of the policy and could be discussed

Response 62: We have discussed the themes regarding viral load results when it was not required by the policy. Please see Line 468 to Line 474

Query 63:2. Drug packaging in fewer bottles vs anonymity may need further explanation for clarity

Response 63: I have added an explanation on the fact that a lot of bottles are associated with making noises in people`s bag which may cause other people to query the contents of their bags. Please see Line 499 to Line 501

Query 64:3. Other potential limitations to consider: patients not included in the study for 1st hand information, study conducted in health facilities with prior 6MMD experience

Response 64: We have added these in the limitations section. Please see Line 521 to Line 523

Conclusion:

Query 65:1. It is not clear from the analysis/discussion to support the conclusion of understanding of policy as one of the major challenges- is it by frequencies in responses/themes?

Response 65: It is by frequencies of the responses/themes. We conducted a coding matrix to determine the frequency of the themes. We have included this in manuscript. Please see Line 188

Minor Observations

Query 66:1. 'Please note it is not acceptable for an author to be the sole named individual responsible for ensuring data access' (https://journals.plos.org/globalpublichealth/s/data-availability#loc-acceptable-data-sharing-methods)

Response 66: We have amended the data access statement. All the data relevant to the article has been included in the article.

Query 67:2. Missing author affiliation, Stella Mahuva

Response 67: That has been added

Query 68:3. Some punctuation, spacing, section and subsection headings formatting required

Response 68: We have formatted the document to show all these changes

Query 69:4. Unpack CSV abbreviation on 1st use in the document

Response 69: This has been done. Please see Line 159

Query 70:5. Statistics and data (STATA) software (unpacking abbreviation)

Response 70: This has been done. Please see Line 174 to Line 175

Query 71:6. ASO abbreviation (unpack for 1st time use)

Response 71: This has been done. Please see Table 1

Sincerely,

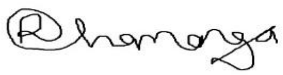

Rachel Chamanga Kanyenda
